# Signal, bias, and the role of transcriptome assembly quality in phylogenomic inference

**DOI:** 10.1186/s12862-021-01772-2

**Published:** 2021-03-16

**Authors:** Jennifer L. Spillane, Troy M. LaPolice, Matthew D. MacManes, David C. Plachetzki

**Affiliations:** 1 Molecular, Cellular, and Biomedical Sciences Department, University of New Hampshire, Durham, NH 03824 USA; 2 Hubbard Center for Genome Studies, University of New Hampshire, Durham, NH 03824 USA

**Keywords:** Phylogenomics, Assembly quality, Phylogenetic signal, Compositional bias, Transcriptomes

## Abstract

**Background:**

Phylogenomic approaches have great power to reconstruct evolutionary histories, however they rely on multi-step processes in which each stage has the potential to affect the accuracy of the final result. Many studies have empirically tested and established methodology for resolving robust phylogenies, including selecting appropriate evolutionary models, identifying orthologs, or isolating partitions with strong phylogenetic signal. However, few have investigated errors that may be initiated at earlier stages of the analysis. Biases introduced during the generation of the phylogenomic dataset itself could produce downstream effects on analyses of evolutionary history. Transcriptomes are widely used in phylogenomics studies, though there is little understanding of how a poor-quality assembly of these datasets could impact the accuracy of phylogenomic hypotheses. Here we examined how transcriptome assembly quality affects phylogenomic inferences by creating independent datasets from the same input data representing high-quality and low-quality transcriptome assembly outcomes.

**Results:**

By studying the performance of phylogenomic datasets derived from alternative high- and low-quality assembly inputs in a controlled experiment, we show that high-quality transcriptomes produce richer phylogenomic datasets with a greater number of unique partitions than low-quality assemblies. High-quality assemblies also give rise to partitions that have lower alignment ambiguity and less compositional bias. In addition, high-quality partitions hold stronger phylogenetic signal than their low-quality transcriptome assembly counterparts in both concatenation- and coalescent-based analyses.

**Conclusions:**

Our findings demonstrate the importance of transcriptome assembly quality in phylogenomic analyses and suggest that a portion of the uncertainty observed in such studies could be alleviated at the assembly stage.

**Supplementary Information:**

The online version contains supplementary material available at 10.1186/s12862-021-01772-2.

## Background

The genomics revolution has resulted in a transformation of the approaches that scientists use to estimate phylogeny by vastly increasing the number of available independent genetic markers [1, 2], as well as the number of taxa included in phylogenetic analyses [3]. However, for taxa that remain largely unrepresented in publicly available datasets, generating a large number of genetic markers, often accomplished as part of a de novo whole genome sequencing project, continues to be a challenge. Transcriptome sequencing is a more accessible method of generating a reduced representation of the nuclear genome that requires fewer sequenced reads and is therefore less expensive than whole genome sequencing (although it is not without its own challenges, see [4]). In addition, transcriptomes perform comparably to genomes in phylogenomic studies when used with robust methods of ortholog identification [5]. For these reasons, data derived from transcriptome assemblies have become widely used in phylogenomic studies and have come to represent a mainstream approach to phylogenetic reconstruction [6–10].

The generation of a phylogenomic data matrix is a complex and critical process, as biases introduced at this point can propagate in downstream analyses in unpredictable ways. Phylogenomic data matrices are composed of multiple (often hundreds of) partitions, alignments of orthologous loci that have been filtered and concatenated together (concatenation-based methods) or analyzed as separate gene trees to inform species trees (coalescent-based methods), resulting in data matrices that are highly dimensional. In addition, phylogenomic datasets are often comprised of an agglomeration of data from multiple research groups that may have leveraged different sequencing and assembly strategies. Therefore it is not surprising that there are still many questions concerning the best practices related to the generation and application of these massive new datasets to phylogenomics [11–13]. Many researchers have addressed questions related to the most appropriate modeling schemes for different partitions of the data matrix [14–19]. Some have considered the impact of incomplete lineage sorting in phylogenomic reconstruction and have leveraged this property of recently diverged lineages to inform species trees [20, 21]. Others have sought to examine differential phylogenetic signal among partitions in order to maximize phylogenomic performance [22, 23]. Increasingly, researchers have added the additional step of recoding the amino acid data matrix in an attempt to account for saturation and compositional heterogeneity ([16, 22–24], although see [25]). While each of these issues is critical to consider in phylogenomic studies, collectively they deal with aspects of the analyses that occur after transcriptome datasets have been assembled. In most cases, biases introduced during the generation of the primary transcriptome assemblies are not explicitly addressed and may persist in influencing downstream inferences.

Whole transcriptome sequencing is itself a relatively new technology, having gained widespread popularity only in the past decade [26]. Therefore, RNA-seq data are commonly treated inconsistently among different phylogenomic studies. While many genomics studies have investigated methodological impacts of read trimming [27, 28], error correction [29–31], different approaches to transcriptome assembly [32], and quality assessment [33–35], researchers using transcriptome assemblies for phylogenomic applications have been slow to adopt many of these recommendations (but see [36–39]). Phylogenomics studies commonly provide few details regarding the nature and quality of the transcriptome assemblies used as input in phylogenomic workflows.

To date there has been no empirical study of how transcriptome assembly quality may affect downstream phylogenomic analyses, although many impacts are possible. Poor-quality assemblies may alter the accuracy of ortholog prediction, alignment quality, and phylogenetic signal. We predicted that in phylogenomic analyses, poor-quality assemblies would result in differences in the number and identity of orthogroups obtained as well as differences in the quality of the partition alignments compared to those from higher-quality transcriptomes. Here we examine the effects of transcriptome assembly quality on these metrics. Our research strategy is to eliminate as many variables that arise from phylogenomic workflows as possible so that we can attribute discrepancies in phylogenomic results to the differences in transcriptome assembly quality. We use a well-characterized quantitative metric (*TransRate* score, see “Methods”; [35]) to evaluate transcriptome assemblies and to systematically construct two separate phylogenomic datasets: one of high quality and one of intentionally low quality. We then perform identical phylogenetic analyses on each dataset, allowing the identification of discrepancies between them and the assessment of their relative phylogenomic performance. We find that high-quality transcriptomes produce larger phylogenomic datasets with partitions that have less alignment ambiguity, weaker compositional bias, and are more concordant with the constraint tree, in both concatenation- and coalescent-based analyses, than datasets derived from low-quality transcriptome assemblies. Our results indicate that a portion of the uncertainty in phylogenomic studies likely stems from issues related to the initial assemblies used in preparing phylogenomic data matrices.

## Results

### Datasets chosen based on TransRate scores have different numbers of transcripts, but show little variation in BUSCO score

**Fig. 1:**
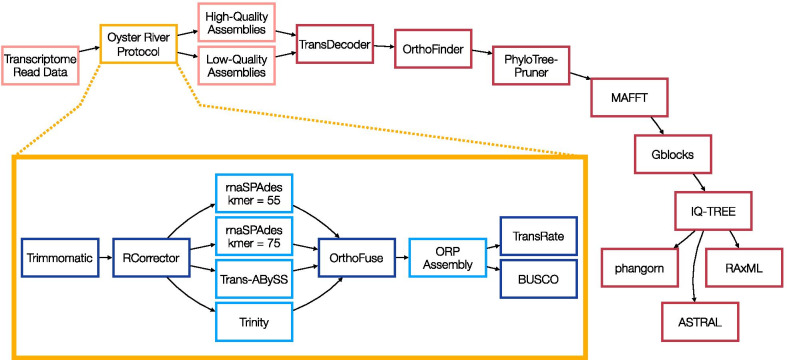
The phylogenomic pipeline used in this analysis from publicly available transcriptomic datasets to partition tree statistics. In the top flowchart red borders indicate bioinformatic tools used while pink ones depict datasets. The Oyster River Protocol is highlighted in yellow, and in the inset: darker blue borders represent steps of the protocol while the resulting transcriptome assemblies are outlined in lighter blue

**Fig. 2:**
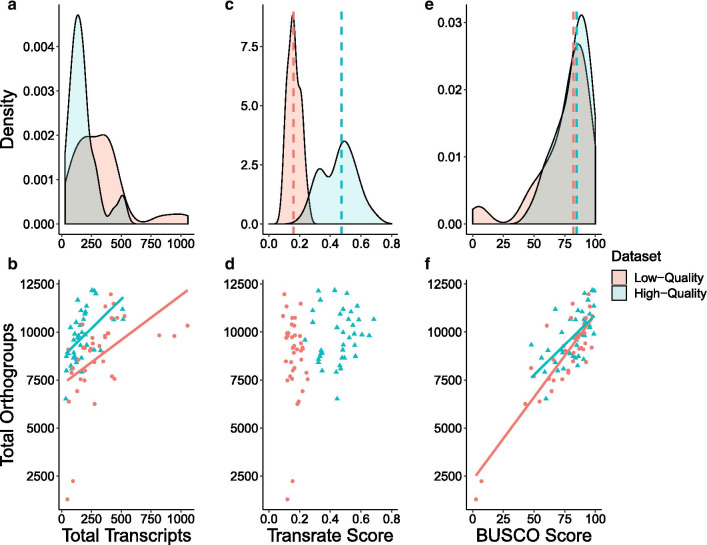
Summary statistics for the high- and low-quality datasets produced. We selected high- and low- quality datasets based on *TransRate* score. This resulted in transcriptome assemblies with both high and low completeness, according to complete *BUSCO* score, in each dataset. Larger assembles in the low-quality dataset did not lead to higher *BUSCO* or *TransRate* scores. Dotted lines in density plots represent medians for each dataset. **a** Density plot of the total number of transcripts (in thousands) in each transcriptome. **b** Relationship between the total number of transcripts (in thousands) and the total number of orthogroups. **c** Density plot of overall *TransRate* scores for each assembly. **d** Relationship between the overall *TransRate* score and the total number or orthogroups. **e** Density plot of complete *BUSCO* score for each transcriptome assembly. **f** Relationship between *BUSCO* score and total number of orthogroups

Our study design controls for several factors that could preclude direct comparison between empirical outcomes in phylogenomic analyses. We focus on the craniate phylogeny because there is little debate about the major relationships within the group and because RNA-seq read data are available from the same tissue type (liver) for a wide range of taxa. The read sets used in this study ranged in size from 13.7 million read-pairs (*Calidris pugnax*) to 46.4 million read-pairs (*Ambystoma mexicanum*). We prepared one high-quality dataset and one low-quality dataset from the same read sets using the Oyster River Protocol (ORP) [32], an assembly pipeline that creates five different transcriptome assemblies for each raw RNA-seq dataset, calculates quality scores for each one, and produces a merged transcriptome assembly consisting of the highest quality unique transcripts (Fig. 1). We leverage the ORP here to intentionally create low-quality transcriptome assemblies that represent real-world empirical outcomes, in addition to high-quality transcriptome assemblies, for each taxon. Reads assembled into significantly fewer transcripts in the high-quality dataset compared to the low-quality dataset (*P* < 0.001, Fig. 2a), with an average of 178,473 and 321,306 transcripts per assembly respectively. The *BUSCO* scores and numbers of orthogroups recovered from orthology analysis of each assembly were both higher on average in the high-quality dataset (Table 1). We compared the number of transcripts in each assembly with the number of orthogroups found for that assembly and identified a significant relationship between these measures in both datasets (linear regression: high-quality dataset, *P* = 0.001; low-quality dataset, *P* = 0.002; Fig. 2b). The high-quality dataset based on overall *TransRate* assembly scores had a median *TransRate* score of 0.47236 (ranging from 0.23542 to 0.68372), while the low-quality dataset’s median *TransRate* score was 0.15943 (ranging from 0.09216 to 0.25281), and overall *TransRate* scores of the two datasets were significantly different from one another (*P* < 0.001; Fig. 2c). We did not find a significant relationship between the overall *TransRate* scores of assemblies and the number of orthogroups obtained for each assembly (linear regression: high-quality dataset, *P* = 0.43; low-quality dataset, *P* = 0.51; Fig. 2d). The number of orthogroups for each dataset was higher in the high-quality dataset, but still largely comparable to the low-quality dataset with the exception of two low-quality read datasets, *Takifugu rubripes* and *Callorhinchus milii*. Each of these datasets recovered much lower numbers of orthogroups than other taxa in the low-quality dataset. In addition to *TransRate* evaluations, the *BUSCO* scores for the low-quality *T. rubripes* and *C. milii* assemblies were also dramatically lower than all other *BUSCO* scores in both datasets (2.7% and 7.2% respectively, compared to the next lowest score: 42.9% for *Notechis scutatus*). However, the overall *BUSCO* scores for the high- and low-quality datasets were not significantly different (Wilcoxon rank sum: *P* = 0.24, Fig. 2e). We observed a significant relationship between *BUSCO* score and number of orthogroups recovered in both datasets (linear regression: high-quality dataset, *P* = 0.001; low-quality dataset, *P* = 0.001; Fig. 2f).

**Table 1:** Read set information and transcriptome assembly metrics

Species	Accession	Read length	NUMBER of reads	High-quality dataset						Low-quality dataset					
				assembler	Number of Transcripts	*BUSCO* complete	*TransRate* score	Orthogroups	Species-specific orthogroups	Assembler	Number of Transcripts	*BUSCO* complete	*TransRate* score	Orthogroups	Species-specific orthogroups
*Alligator mississippiensis*	SRR629636	100	36,130,137	ORP	287,695	91.1	0.50848	12,004	32	SPAdes75	466,618	80.2	0.16986	10,737	20
*Ambystoma mexicanum*	SRR5341572	101	46,417,978	ORP	209,702	98.7	0.57581	11,350	59	SPAdes75	528,158	97.4	0.09216	10,832	61
*Anas platyrhynchos*	SRR7127376	101	20,486,658	ORP	142,201	91.1	0.65376	9813	8	SPAdes75	244,848	86.8	0.2212	9129	11
*Anolis carolinensis*	SRR391653	101	17,152,427	Trans-ABySS	40,327	86.2	0.33263	8729	9	Trinity	56,207	90.1	0.18273	9093	9
*Astyanax mexicanus*	SRR2045431	100	32,893,691	ORP	110,132	98.7	0.55641	9902	44	SPAdes75	180,139	97	0.21902	9187	36
*Balaenoptera acutotostrata*	SRR919296	100	23,923,194	ORP	200,511	89.2	0.53496	11,048	10	SPAdes75	364,918	86.1	0.15086	9729	14
*Bufo bufo*	ERR1331718	126	37,410,097	Trans-ABySS	135,770	94	0.33512	11,671	57	SPAdes75	413,473	94.4	0.10086	11,968	34
*Caecilia tentaculata*	SRR5591453	101	28,784,422	ORP	107,413	81.8	0.56427	8737	35	SPAdes75	196,546	77.9	0.15651	7993	29
*Caiman crocodilus*	ERR2198478	variable	31,864,053	Trans-ABySS	163,595	85.8	0.28529	11,113	3	SPAdes75	436,573	81.5	0.20671	11,475	6
*Calidris pugnax*	ERR1018151	150	13,725,659	Trinity	78,074	85.5	0.46221	8239	10	SPAdes75	83,535	77.9	0.24439	7880	2
*Callorhinchus milii*	SRR513760	76	35,000,000	ORP	124,415	67.3	0.32314	8418	17	SPAdes75	95,463	7.2	0.15425	2232	13
*Canis lupus familiaris*	ERR1331673	100	36,371,999	ORP	437,158	83.8	0.58601	10,633	3	SPAdes75	819,785	86.5	0.1697	9826	10
*Dasypus novemcinctus*	SRR494766	101	31,705,473	Trans-ABySS	55,634	79.2	0.33783	8868	7	SPAdes75	192,657	66	0.12049	7478	6
*Felis catus*	ERR1331679	100	40,228,662	ORP	516,209	79.5	0.51854	10,659	8	SPAdes75	945,952	85.2	0.20215	9790	4
*Gadhus morhua*	SRR2045420	100	18,943,673	ORP	85,927	74	0.46787	8082	29	SPAdes75	131,171	64.3	0.21936	6919	15
*Gallus gallus*	ERR1298598	100	14,955,711	ORP	272,485	72.3	0.48137	9069	8	SPAdes75	444,042	62.1	0.15068	7562	9
*Haplochromis burtoni*	SRR387451	101	16,142,312	Trans-ABySS	40,240	69.3	0.34653	7981	14	SPAdes75	60,824	54.5	0.19438	6379	48
*Homo sapiens*	SRR5576267	101	20,633,201	ORP	171,048	72.6	0.48352	9971	5	SPAdes75	317,048	74.2	0.16465	9271	8
*Ictalurus punctatus*	SRR917955	100	28,319,586	ORP	99,232	83.8	0.49223	8645	32	SPAdes75	159,608	73	0.25281	7538	34
*Latimeria menadoensis*	SRR576100	109	39,788,120	Trans-ABySS	101,337	73.3	0.34696	8913	69	SPAdes55	258,443	82.2	0.11311	9692	13
*Lepidophyma flavimaculatum*	DRR034613	variable	20,350,517	Trans-ABySS	121,895	91.4	0.28563	10,505	59	SPAdes75	174,935	90.7	0.14923	10,395	37
*Lepisosteus oculatus*	SRR1287992	101	22,992,842	Trans-ABySS	75,239	95.4	0.44361	10,235	55	SPAdes75	195,782	88.5	0.15598	9172	42
*Lethenteron camtschaticum*	SRR3223459	125	29,559,367	Trans-ABySS	125,856	90.4	0.32322	8577	292	SPAdes75	274,262	92.4	0.14484	8431	93
*Lissotriton montandoni*	SRR3299753	100	32,548,205	ORP	195,142	95.4	0.46462	10,934	68	SPAdes75	387,445	91.1	0.12708	9939	56
*Notamacropus eugenii*	DRR013408, DRR013409, DRR013410	100	24,378,361	ORP	198,447	88.5	0.60651	9859	24	SPAdes75	347,172	82.2	0.16235	8917	28
*Notechis scutatus*	SRR519122	90	25,626,764	ORP	168,738	58.7	0.43875	7908	31	SPAdes75	277,137	42.9	0.18596	6254	32
*Oophaga sylvatica*	SRR9120851	100	22,858,029	ORP	166,747	56.1	0.47685	8650	18	SPAdes75	423,029	49.8	0.13789	7690	24
*Oryctolagus cuniculus*	ERR1331669	100	22,037,691	ORP	158,880	84.8	0.5591	9304	4	SPAdes75	349,879	81.8	0.11102	8469	5
*Parus major*	SRR1847228	101	35,000,000	ORP	155,826	95	0.54739	10,349	16	SPAdes75	261,539	91.7	0.20877	9408	18
*Pelodiscus sinensis*	SRR6157006	150	24,740,727	Trinity	274,343	99	0.32231	12,143	40	SPAdes75	367,085	95.4	0.11519	11,332	23
*Pelusios castaneus*	SRR629649	100	45,163,324	ORP	254,815	97.4	0.42891	12,168	31	SPAdes75	419,831	92.4	0.15182	10,728	19
*Protopterus* sp.	ERR2202465	150	18,298,224	SPAdes75	327,343	61.4	0.34033	9036	127	Trinity	141,824	65.7	0.21121	8558	89
*Rana pipiens*	SRR1185245	101	35,791,829	ORP	136,439	82.2	0.52391	9695	36	SPAdes75	238,110	75.4	0.20868	9029	16
*Rhinella marina*	SRR6311453	100	27,446,915	ORP	511,551	67.4	0.48377	11,184	48	SPAdes75	1,056,698	60.4	0.16511	10,330	32
*Rhinolophus sinicus*	SRR2273875	101	30,559,494	ORP	184,384	90.8	0.68372	10,658	14	SPAdes75	392,613	86.2	0.1185	9933	9
*Squalus acanthias*	ERR1525379	Variable	35,000,000	Trans-ABySS	101,153	84.5	0.23542	9582	25	SPAdes75	363,863	83.2	0.12189	9803	79
*Takifugu rubripes*	SRR1005688	76	35,796,911	Trans-ABySS	35,375	59.7	0.44456	6518	13	SPAdes75	48,271	2.7	0.1206	1287	22
*Trachemys scripta*	ERR2198830	150	22,741,770	SPAdes75	210,713	47.8	0.48531	9322	6	Trinity	94,129	47.6	0.16631	8123	3

For each species, we assembled the transcriptomic reads using the Oyster River Protocol. Of the five resulting transcriptome assemblies, we chose the one with the highest overall *TransRate* score and the one with the lowest overall *TransRate* score to use in the high- and low-quality datasets, respectively. We also quantified the number of transcripts in each assembly, calculated the complete *BUSCO* score, and inferred orthogroups using *OrthoFinder*

### High-quality assemblies result in a larger number of partitions after processing

**Fig. 3:**
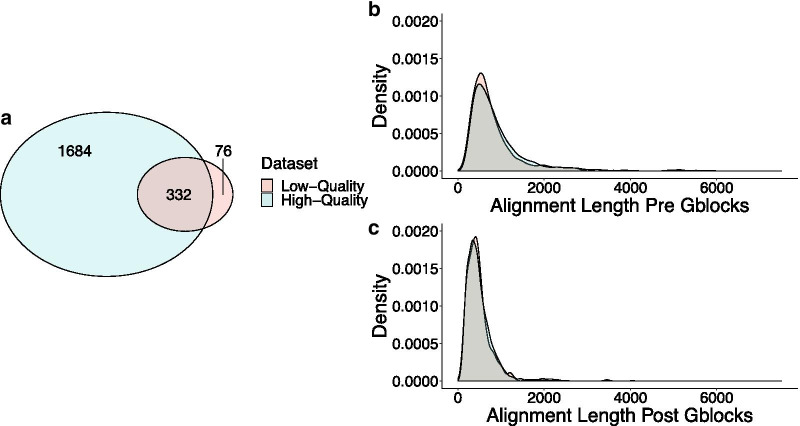
Length of alignments and number of partitions for each dataset. **a** Venn diagram showing number of partitions unique to each dataset, and common between them. The number of partitions recovered through the phylogenomic analysis pipeline is fivefold higher when the dataset is made up of high-quality transcripts compared to lower-quality ones. **b** Density plot of alignment lengths of each partition before filtering with *Gblocks*. **c** Density plot of alignment lengths of each partition after filtering with *Gblocks*. While the lengths of the individual alignments are significantly different before *Gblocks* filtering, they are similar afterwards

Next, we isolated one-to-one orthologs that were present in 100% of taxa. After aligning and filtering these orthologs into partitions we observed that one major impact of assembly quality on phylogenomic data matrix construction is the scale of the resulting data. We obtained 2016 data partitions from the high-quality dataset, whereas we recovered only 408 data partitions from the low-quality dataset. 332 data partitions in both the high- and low-quality datasets included an identical reference sequence from the *Mus musculus* reference transcriptome, demonstrating that a majority of the data partitions recovered from the low-quality dataset are also represented in the high-quality dataset (Fig. 3a). The high-quality dataset however, included many more unique sequence partitions (1684 unique partitions compared to 76, Fig. 3a). The distributions of alignment lengths between datasets differed significantly before alignment filtering (Wilcoxon rank sum, *P* = 0.02; Fig. 3b) with alignments in the high-quality dataset being longer on average, but not after alignment filtering (Wilcoxon rank sum, *P* = 0.79; Fig. 3c).

### High-quality alignments possess reduced compositional bias and alignment ambiguity

**Fig. 4:**
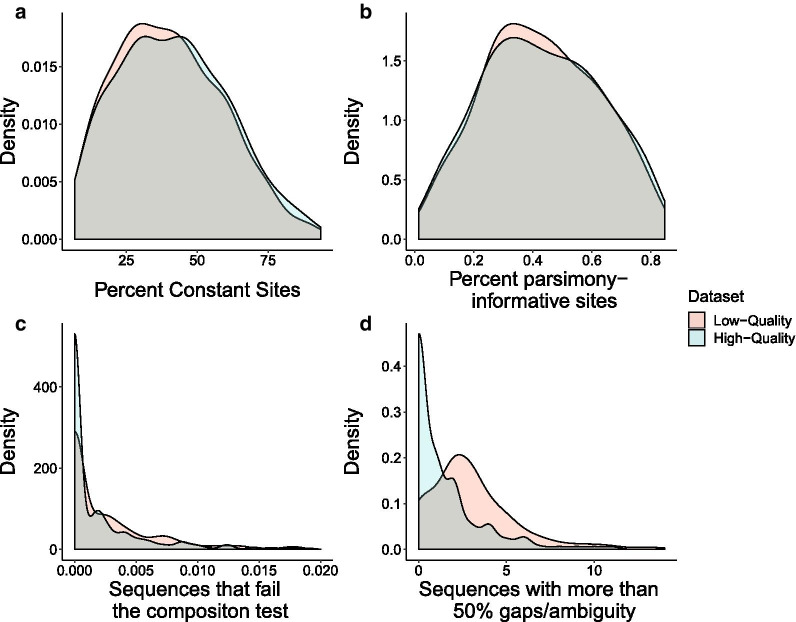
Density plots of four alignment metrics for both datasets. Alignments created from low-quality transcriptome assemblies have similar percentages of constant and parsimony-informative sites, but higher compositional bias and ambiguity when compared to alignments from high-quality assemblies. **a** Percentage of constant sites in each partition alignment. **b** Percentage of parsimony-informative sites in each partition alignment. **c** Number of sequences that fail the composition test, normalized by partition alignment length. **d** Number of sequences that contain more than 50% gaps/ambiguity in each partition alignment

In order to draw direct comparisons between the partitions derived from the high- and low-quality datasets, we examined the alignment statistics of the 332 partitions that were shared between them. The percentage of constant sites in each alignment was not significantly different between the high- and low-quality datasets (Wilcoxon rank sum, *P* = 0.37, Fig. 4a). Similarly, the percentage of parsimony-informative sites in the alignments did not differ significantly between the two datasets (Wilcoxon rank sum, *P* = 0.89, Fig. 4b). However, the number of sequences that failed the composition Chi^2^ test [40] and the number of sequences with over 50% alignment ambiguity were significantly different between the two datasets (composition—Wilcoxon rank sum, *P* = 0.006, Fig. 4c; ambiguity—Wilcoxon rank sum, P < 0.001, Fig. 4d), and both of these metrics were higher in the low-quality dataset.

### No bias in gene content in partitions from both high- and low-quality datasets

Phylogenetic information content of a given phylogenomic data matrix could be impacted if the partitions themselves are drawn from a biased set of loci. In order to understand the genetic composition of phylogenomic datasets derived from high- and low-quality assemblies, we conducted gene ontology (GO) analysis of the recovered partitions. We did not observe enrichment for functional category in either the high- or low-quality datasets.

### Partitions from high-quality assemblies recapitulate the constraint tree to a larger extent than those from low-quality assemblies in both concatenation- and coalescent-based analyses

**Fig. 5:**
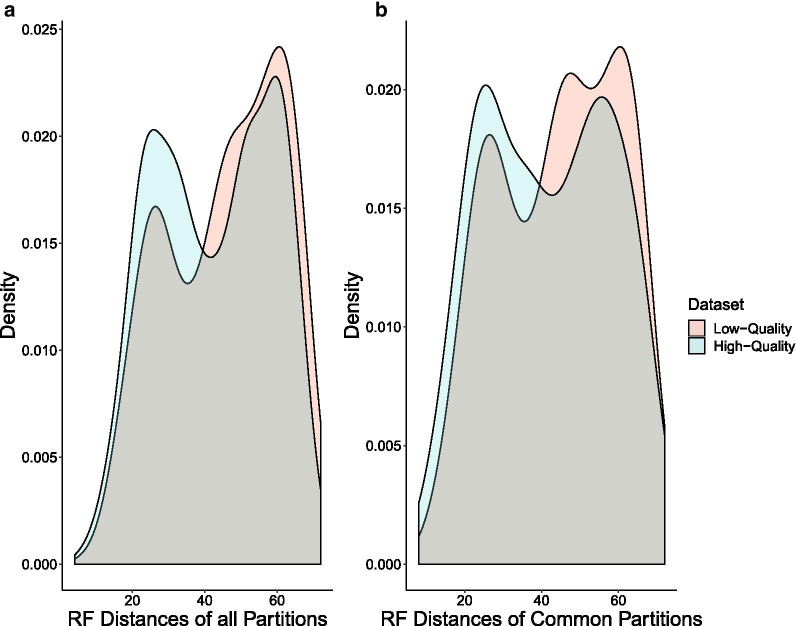
Per partition Robinson–Foulds (RF) distances to the constraint tree are significantly shorter in the high-quality dataset compared with the low-quality dataset. **a** Density plot for all partitions from both datasets. **b** Density plot for only those 332 partitions that are shared between the two datasets

**Fig. 6:**
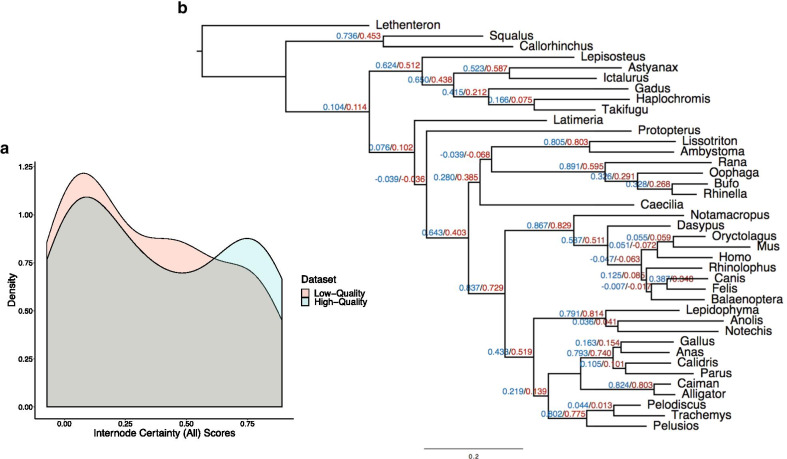
Partitions derived from the high-quality dataset have higher internode certainty all (ICA) values than those derived from the low-quality dataset when compared to the constraint tree. **a** Density plot of ICA values. **b** Average ICA values for each node. Blue represents the high-quality dataset, red represents the low-quality dataset. Negative ICA values suggest that the node conflicts with at least one other node that has a higher support

**Fig. 7:**
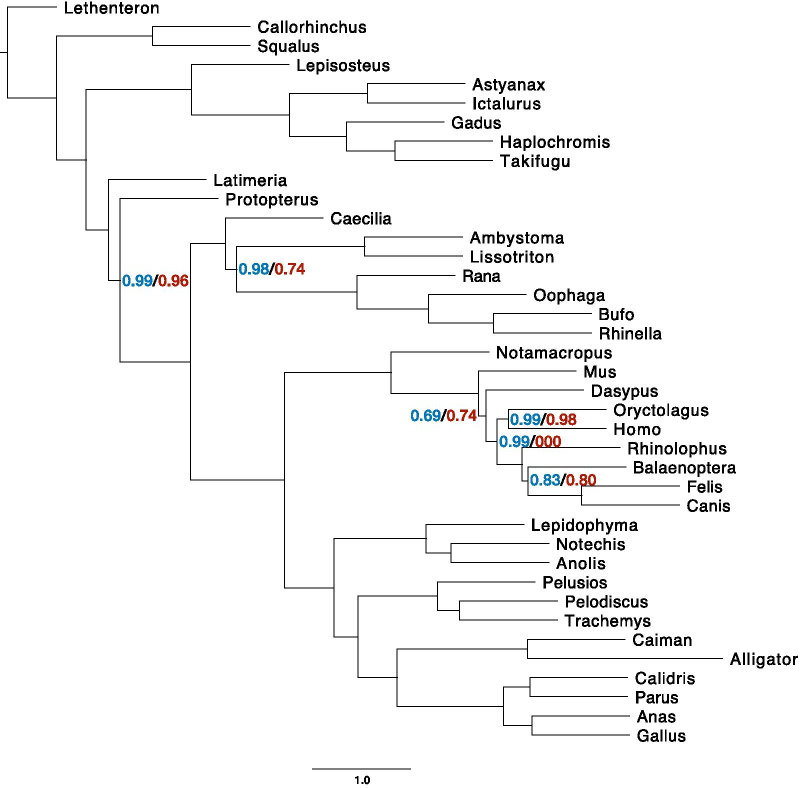
Species tree analysis in *ASTRAL* reveals a similar pattern to concatenation analyses. *ASTRAL* analyses of gene trees from 332 shared partitions from the high- and low-quality datasets result in identical topologies. In addition to normalized quartet scores being higher for gene trees derived from the high-quality dataset, node support values for the high-quality dataset are marginally stronger than those from the low-quality dataset. Support values represent support for quadripartitions of the tree, and only those that were less than 1 are represented

Finally, we sought to understand the impact of assembly quality on phylogenetic signal. We first compared the two datasets to a constraint tree representing the current view of craniate relationships [41, 42] by using Robinson–Foulds (RF) distances and internode certainty all (ICA) values in concatenation analyses. RF distances reflect topological differences between partition subtrees and the constraint tree [43], whereas ICA values indicate the proportion of data partitions for the high-quality and low-quality datasets that support each node in our constraint tree [44]. We found that the high-quality dataset had significantly lower RF values overall than the low-quality dataset (Wilcoxon rank sum, *P* < 0.001; Fig. 5), indicating a shorter distance to the constrained craniate tree for the partitions in the high-quality dataset. The partitions derived from the high-quality dataset possessed characteristically higher ICA values than those from the low-quality dataset, although the distributions of scores were not significantly different (Wilcoxon rank sum, *P* = 0.47; Fig. 6) likely due to low statistical power. We also investigated the relative performance of the two datasets in coalescent-based analyses using *ASTRAL* [20, 45]. Similarly, we found that the high-quality dataset produced gene trees with less discordance to the estimated species tree than their low-quality counterparts, with a normalized quartet score of 0.75 for the high-quality partitions compared to 0.73 for the low-quality partitions. Both datasets resolved the same topology in *ASTRAL* analyses (Fig. 7).

In summary, we find that datasets derived from high-quality transcriptome assemblies yield larger phylogenomic matrices than those from low-quality transcriptome assemblies. In addition to being more numerous, the data partitions in the high-quality dataset are also less compositionally biased, have less alignment ambiguity, and are less discordant with the constraint tree.

## Discussion

Given the ubiquity of transcriptome usage phylogenomics, we sought to understand how sub-optimal data handing practices during the assembly process may affect downstream phylogenomic analyses. We observed a general trend in our analyses where more accurate transcriptome assemblies resulted in phylogenomic datasets with a greater number of unique data partitions, longer alignments, fewer ambiguous regions, less compositional bias, greater consistency with the known phylogeny in concatenation-based analyses, and higher normalized quartet scores in coalescent-based analyses. We did not uncover any functional biases in the GO terms associated with either dataset.

### High-quality assemblies result in a larger number of partitions after phylogenomic processing

The most dramatic difference between the high- and low-quality phylogenomic data matrices is the number of orthogroups that contained all species. After estimating one-to-one orthologs, aligning the orthologs, and filtering the alignments, this difference led to ~ five times the number of data partitions in the high-quality dataset compared with the low-quality dataset. Transcriptomic assembly errors that are expected to pervade low-quality assemblies include the generation of chimeric transcripts, the generation of incomplete transcripts, or the failure to generate transcripts due to missing data [32, 35]. Our results from analyses of the low-quality assemblies indicate that incompletely assembled transcripts may be at least partially responsible for the differences in partition number because the partition alignments before filtering are significantly longer in the high-quality dataset, indicating fewer incompletely assembled transcripts in the latter. While *OrthoFinder* [46, 47] may be somewhat robust to these issues, when more complete sequence information is provided in high-quality transcripts, *OrthoFinder* analyses identify significantly greater numbers of orthogroups that contain a high proportion of species and therefore greater numbers of orthologs. Missing transcripts could also impact the accuracy of downstream analyses and the establishment of one-to-one orthologs because, depending on what data are missing, orthologs and paralogs could become conflated between taxa. Our results are consistent with this expectation because among partitions that are shared between high- and low-quality datasets, those from the high-quality dataset show more accurate phylogenetic signal, as measured by constraint tree analyses in concatenation analyses and in coalescent approaches (see below).

We identified two transcriptome assemblies within the low-quality dataset, *Takifugu rubripes* and *Callorhinchus milii*, which have dramatically lower *BUSCO* scores and number of orthogroups recovered than other taxa within the same dataset. We included these two taxa in the analysis despite their extreme *BUSCO* scores for a number of reasons. First, these taxa occupy important phylogenomic positions within the craniate tree and publicly available craniate liver transcriptome datasets are somewhat limited. Second, while the *TransRate* scores for these two taxa are below average for the low-quality dataset (Fig. 2c, d), they are well within the distribution of low-quality assembly *TransRate* scores, indicating that these two taxa yield assemblies that are contiguous and correctly assembled to a comparable extent to the other assemblies included in that dataset. While it is standard practice to deposit raw reads into public databases, the read-sets for these two species appeared to have been trimmed prior to public data deposition [48], making them shorter than the other read-sets. We identified average read length as the probable reason for the lack of genic completeness as measured by *BUSCO* for these two taxa. Due to this shorter read length, these two organisms performed especially poorly in *rnaSPAdes* with a kmer length of 75 (only reads of length k + 1 are used in assembly), which was subsequently the assembly used in the low-quality dataset for both of these organisms. Importantly, these two species’ corresponding assemblies in the high-quality dataset were not outliers (Fig. 2c, d), indicating that a robust assembly strategy can compensate for sub-optimal sequence reads. Therefore, by including these two taxa, we were able to represent a situation commonly encountered in phylogenomic studies that utilize publicly available data—the inclusion of reads of poor quality or that have been previously processed.

The drastic difference in number of partitions in the low-quality dataset compared to the high-quality dataset is due in part to these two taxa having smaller and less complete assemblies than all others. However, when we relax the strict filtering to include orthogroups with up to two missing taxa (thereby giving the low-quality dataset the opportunity to exclude *T. rubripes* and *C. milii*) we find that the high-quality dataset still has over 1600 more partitions than the low-quality dataset, and therefore the inclusion of these taxa is not the only driving force behind the difference in partitions between the datasets. While there are fewer partitions in the low-quality dataset, it is still a sufficient number (408) for most downstream phylogenomic applications. Therefore, we conclude that while the situation encountered with the *T. rubripes* and *C. milii* RNA-seq data has an effect on some aspects of our phylogenomic analysis, their effects are only manifested in analyses of the low-quality assemblies and extend beyond data drop out.

### Low-quality assemblies produce alignments with more compositional bias and alignment ambiguity than high-quality assemblies

In the process of making gene trees for each of the data partitions, *IQ-TREE* calculates a number of metrics about the partition alignments and the sequences within them [40]. One such test is for compositional homogeneity, which measures the character composition of amino acids in each sequence against the character composition in the whole alignment. Here, we chose to assess changes in compositional heterogeneity using the simple Chi^2^ test implemented in *IQ-TREE* [40, 49]. Heterogeneity or bias in amino acid composition can mislead phylogenetic inferences: distantly-related organisms that have high compositional bias may erroneously group together [50]. The number of sequences failing the composition test—that is, the number of sequences with higher compositional heterogeneity than expected by chance—was higher in the partitions from the low-quality dataset. Because these partitions have direct counterparts in the high-quality dataset, this difference in compositional heterogeneity is directly attributable to a difference in assembly quality. Similarly, the partitions from the low-quality dataset also contained more sequences with over 50% gaps or ambiguity in the alignment. While global alignments often contain gaps because of insertions or deletions in the sequences, comparison of the two datasets implies that the greater number of gaps in the low-quality dataset also results from incorrect transcriptome assemblies rather than natural variation.

The low-quality dataset contained some partitions that the high-quality dataset did not have. These partitions could be unique transcripts only assembled in the low-quality dataset, or they could be the result of differential pruning of paralogous sequences between the two datasets, resulting in a different *Mus* identifying sequence in two partitions that represent the same gene family. They might also be erroneous or duplicate partitions that were misidentified during the *OrthoFinder* procedure as separate gene families due to poor assembly quality. In principle, differential data assembly quality could inject bias into the resulting orthogroups if some loci, perhaps short or highly expressed genes, were preferentially assembled among the different datasets, however our GO analyses showed no enrichment or depletion of GO terms in these partitions.

### Partitions derived from high-quality assemblies perform better in both concatenation- and coalescent-based phylogenomic analyses

In this study, we used quantitative analyses to assess phylogenomic performance of the high- and low-quality transcriptome assemblies. We showed that the individual partitions included in the high-quality dataset were closer to the constraint tree by calculating RF distances. The high-quality dataset had significantly smaller RF distances to the constraint tree in concatenation-based analyses (Wilcoxon rank sum, *P* < 0.001) and less discordance in coalescence-based analyses as indicated by normalized quartet score (Fig. 7). While the ICA values of the high-quality dataset were not significantly higher than those in the low-quality dataset, the trend shows that ICA values are generally higher among partitions from the high-quality dataset with a greater proportion of partitions falling above 0.6. This indicates that the gene trees estimated from the high-quality dataset partitions are more consistent with the constraint tree of craniates and show greater phylogenetic signal [51] than the low-quality dataset in concatenated analyses (Fig. 6b).

### Limitations in data availability and statistical power do not affect our conclusions

Our research strategy was to eliminate as many variables as possible so that we could isolate the effects of assembly quality on phylogenomic performance. These variables include the type of tissue that RNA-seq datasets are derived from and the topology itself. We treat the craniate phylogeny, for which few arguments remain regarding the relationships of the taxa included [41, 42], as a “known” parameter to constrain our analyses. In this way we were able assess how close a given analysis accords with that constraint in light of other perturbations like assembly quality. However, it is notable that phylogenomic trees based on the 332 data partitions that are common to both the high-quality and low-quality datasets, using either concatenation- or coalescent-based methods, fail to resolve the craniate phylogeny accurately (Fig. 7; Additional file 2: Figure S1). While this result has no bearing on any of the conclusions presented here, it is likely due to two factors. First, the magnitude of both datasets, 332 partitions, is far fewer than that included in recent well-resolved phylogenomic studies of craniates [41]. Here, our utilization of only 332 partitions derives from the necessity that they be shared between the high- and low-quality assemblies, and therefore directly comparable. Second, our taxon sampling is low compared to recent phylogenomic studies of craniates. This is due to the requirement of our study design that RNA-seq reads be derived from a homologous tissue (e.g. liver) across taxa, offering a different type of direct comparison. While we were able to represent most of the major lineages of craniates with RNA-seq data derived from liver tissue, it was not possible to provide greater taxon sampling given current publicly available data while also preserving taxonomic evenness in sampling across various vertebrate clades.

We also point out that some of the quantitative measures reported here (e.g. ICA) show clear trends that favor the high-quality dataset over the low-quality dataset but are not significantly different. This may be due to intrinsic differences in statistical power that make it unlikely that a significant difference would be identified between datasets for those measures that have fewer data points (RF distances yield one data point per gene tree (332) while ICA scores provide one data point per node [34]). However, we do not observe a single instance of the low-quality dataset being quantitatively or qualitatively better than the high-quality dataset in terms of phylogenetic signal for any of our measures.

### Conclusions

Phylogenomic approaches leverage great power to resolve phylogenetic relationships, but they also include many analytical pitfalls associated with ortholog identification, alignment filtering, and model selection. While these pitfalls have been well-characterized, we chose to focus on transcriptome assembly quality—a more fundamental and largely overlooked aspect of phylogenomic analyses. We addressed this problem empirically using a study design that controls for variables including taxon selection, data type, data provenance, and phylogenetic uncertainty. We show that assembly quality, when all other factors are controlled, can have a dramatic impact on phylogenomic analyses in three ways. First, the richness and size of the dataset can differ profoundly when assembly errors are prevalent in the data. Second, alignments created from low-quality assemblies are more prone to ambiguity and compositional bias than their high-quality counterparts. And third, the partitions derived from high-quality assemblies have greater phylogenetic signal to resolve true evolutionary relationships than partitions derived from low-quality assemblies. We conclude that additional analytical interventions aimed at improving assembly quality, such as the Oyster River Protocol [32], are likely worth the additional effort.

## Methods

### Read selection and assembly

To understand the effects of transcriptome assembly quality on phylogenomic inference, we created two datasets, one of high and one of low quality, from publicly available transcriptomic reads (see Additional file 1 for more information on data availability). All read data are available on the European Nucleotide Archive (Table 1). We focused on craniates because there are few remaining disputes on the craniate phylogeny [41] and these well-established phylogenetic relationships serve as a comparison to the topologies found using our high- and low-quality transcriptome assemblies. Our research strategy was to assemble high- and low-quality transcriptomes from the same set of reads. We obtained Illumina-generated paired-end liver transcriptomic reads for 37 vertebrate species spanning the majority of the diversity contained within the clade as well as one craniate outgroup. We assembled each read set using the Oyster River Protocol (ORP) version 2.2.3 [32] on a Linux computer with 24 CPUs and 128 GB of RAM. In brief, this protocol begins by adapter- and quality-trimming reads using *Trimmomatic* version 0.38 [52] as per recommendations in MacManes [27], after which it corrects read errors using *Rcorrector* version 1.0.8 [30] following recommendations from MacManes and Eisen [29]. The ORP then assembles trimmed and corrected reads using three different assemblers: *Trinity* version 2.8.5 [53] with a kmer length of 25, *Trans-ABySS* version 2.0.1 [54] with a kmer length of 32, and *rnaSPAdes* version 3.14 [55] using kmer lengths of 55 and 75. The protocol continues by merging the resultant four assemblies and clustering them into isoform groups. The ORP then scores all transcripts using *TransRate* version 1.0.3 [35] which maps the read sets onto the assembly and, based on the mapping, detects assembly errors such as fragmentation, chimerism, and local misassembly. *TransRate* then uses this error information to assign quality scores to each transcript before integrating these individual scores into a score for the assembly as a whole. The ORP selects the member of each isoform group with the highest *TransRate* score and places it into a new file. Finally, the protocol uses *cd-hit-est* version 4.8.1 [56] and a 98% sequence identity threshold to reduce transcript redundancy. The assemblies produced by the ORP are therefore populated by the highest quality, non-redundant sequences produced by any of the five possible assembly strategies [32]. A graphical summary of this protocol and our phylogenomic pipeline can be found in Fig. 1.

### Quality analysis and high- and low-quality dataset construction

We evaluated each of the five assemblies generated from the ORP (from *Trinity*, *TransABySS*, *rnaSPAdes* at two kmer lengths, and the final ORP assembly) for each species in two main ways. We used *BUSCO* version 3.0.1 [57], which uses benchmarking universal single copy orthologs to measure the genic completeness of an assembly. In addition, because we were primarily interested in assessing the structural differences in the transcriptome assemblies arising from errors during the assembly process, we generated *TransRate* scores for each assembly. Of the five assemblies for each species, we chose the assembly with the highest overall *TransRate* score to be part of the high-quality dataset, and the one with the lowest overall score to be part of the low-quality dataset. We selected assemblies for each dataset regardless of which assembler produced them, resulting in datasets that contain transcriptomes from multiple different programs. This was done in part to simulate transcriptomic datasets in other studies that may be constructed from preexisting transcriptome assemblies, rather than those that have reassembled each dataset using the same program and to provide appropriate contrast between the high- and low-quality datasets. We performed all subsequent steps on both datasets in parallel.

### Orthogroup inference, statistics, and data partition creation

We used *TransDecoder* version 5.5.0 [58] to translate all transcript sequences to amino acid sequences. The transcriptome assembly process assigns each new transcript a unique name so that it can be differentiated within the assembly. This means that the high- and low-quality assemblies do not share identical transcripts or names common to both assemblies, making the direct comparison of sequences impossible. To circumvent this issue, we added the *Mus musculus* reference transcriptome (release 96) [59] to both datasets just before the *TransDecoder* step so that a *Mus* sequence would be present in many orthogroups and partitions downstream. This created a common naming system by which we could compare the content of orthogroups and partitions derived from assemblies of high and low quality later in the analysis.

For each dataset (containing either the high-quality or low-quality transcriptome assemblies for the 38 craniate species plus the *Mus* reference transcriptome) we performed a separate *OrthoFinder* version 2.3.3 analysis [46, 47]. We then used linear regressions in *R* version 3.5.2 [60] to evaluate the relationship between the total number of orthogroups found for each taxon and three other measures: the total number of transcripts in each assembly, the overall *TransRate* score, and the *BUSCO* complete score. We also plotted the distributions of these three measures for each dataset and performed Wilcoxon rank sum tests in *R* to determine if they were statistically different.

We filtered the resulting orthogroups so that we retained only those that had each taxon represented by at least one sequence. From these, we obtained one-to-one orthologs using *PhyloTreePruner* [61]. We realigned these sequences using *MAFFT* version 7.305b using the “auto” setting [62], and filtered the alignments for poorly aligned or divergent regions using *Gblocks* version 0.91b [63, 64] with options “− b2 = 0.65 − b3 = 10 − b4 = 5 − b5 = a” in the script “gblocks_wrapper.pl” [65]. Finally, we concatenated all sequences into a NEXUS file for each dataset. We measured the lengths of the alignments both before and after *Gblocks* and compared the content of both groups of partitions by using the *Mus* sequence headers as common identifiers that were present in both datasets and determined the numbers of unique and shared partitions. We then used *IQ-TREE* version 1.6.12 under the LG model [40] to find individual gene trees for each partition in each dataset.

### GO analysis and alignment metrics

To investigate the differences in content and qualities of the partitions between the two datasets, we separated the partitions into groups containing only those that were unique to each dataset, and only those that were shared between the two datasets. We used *InterProScan* version 5.31–70.0 [66] to annotate the partitions unique to each dataset and then performed a gene ontology (GO) analysis with *topGO* version 2.32.0 [67] in *R* version 3.5.2 [60] to check for any functional enrichment or depletion bias in the partitions of either dataset. For each partition common to both datasets, we extracted various alignment metrics from the log and information files generated while making partition trees in *IQ-TREE*. These included percent constant sites, percent parsimony-informative sites, number of sequences that failed the Chi^2^ composition test (which we normalized by alignment length), and the number of sequences that contained more than 50% gaps or ambiguity. To test for significant differences, we performed Wilcoxon rank sum tests in *R* version 3.5.2 [60] between the two datasets for each of these measures.

### Constraint tree and comparisons of partition trees

The phylogenetic relationships among the 38 craniate species for which we obtained liver RNA-seq data are well-supported by previous work [41]. Therefore, we used a tree that reflects the most well-supported hypothesized relationships for comparison against the partition trees. Using *Mesquite* version 3.6 [68], we constructed a constraint tree that reflects the widely accepted topology for craniates. We used the high-quality dataset NEXUS alignment file along with this topology to estimate the constraint tree topology with branch lengths in *IQ-TREE* using the LG model [40]. We calculated RF distances [43] from the partition trees in each dataset to the constraint tree using *phangorn* version 2.5.5 [69] in *R* version 3.5.2 [60]. This metric measures the differences in topology (RF distance) from the partition trees to the constraint tree, with smaller numbers indicating less conflict between the two trees. We also calculated ICA values between the individual partition trees and the constraint tree using *RAxML* version 8.2.11 [70]. The ICA refers to the degree of certainty for each internal node of the tree compared to the constraint tree when all other conflicting bipartitions are taken into account for that dataset. Numbers close to 1 show a lack of conflict between the partition tree and the constraint tree [44]. We tested for significant differences between the two dataset distributions using a Wilcoxon rank sum test in *R* version 3.5.2 [60] for both RF distances and ICA values. Finally, we created species trees using the 332 gene trees that were common to both the high-quality and low-quality datasets with a coalescent method implemented in *ASTRAL* version 5.7.4 [20, 45]. We calculated the normalized quartet score for each tree, which represents the percentage of quartet trees in the input trees that are satisfied by the species tree and ranges from 0–1, with higher numbers indicating less discordance.

## Supplementary Information

**Additional file 1: Table S1**. Accession numbers and associated studies of RNA-seq read sets used in these analyses.

**Additional file 2: Figure S1.** Phylogenetic trees created using the 332 data partitions shared between the two datasets and concatenation methods do not resolve the accepted craniate phylogeny but produce differing topologies. The trees were built in IQ-TREE using an LG model and nodes are labeled with ultrafast bootstrap approximated branch supports using the “-bnni” (a hill-climbing nearest neighbor interchange search) to reduce the impact of severe model violations. A: Phylogenetic tree for the low-quality dataset. B: Phylogenetic tree for the high-quality dataset.

## Data Availability

The transcriptome assemblies generated and analyzed in this study are available on the Zenodo site, https://doi.org/10.5281/zenodo.3939160 [71]. All custom scripts written for or used in this work as well as commands for programs run are accessible via the GitHub repository, http://github.com/jls943/quality_review [72].

## Abbreviations

ORP: Oyster River Protocol
RF distances: Robinson–Foulds distances
ICA values: Internode certainty all values
GO: Gene ontology

## References

[1] Dopazo H, Santoyo J, Dopazo J (2004). Phylogenomics and the number of characters required for obtaining an accurate phylogeny of eukaryote model species. Bioinformatics.

[2] Blair JE, Ikeo K, Gojobori T, Hedges SB (2002). The evolutionary position of nematodes. BMC Evol Biol.

[3] Dunn CW, Hejnol A, Matus DQ, Pang K, Browne WE, Smith SA (2008). Broad phylogenomic sampling improves resolution of the animal tree of life. Nature.

[4] Vijay N, Poelstra JW, Kunstner A, Wolf JBW (2013). Challenges and strategies in transcriptome assembly and differential gene expression quantification. A comprehensive in silico assessment of RNA-seq experiments. Mol Ecol.

[5] Cheon S, Zhang J, Park C (2020). Is phylotranscriptomics as reliable as phylogenomics?. Mol Biol Evol.

[6] Chen X, Zhao X, Liu X, Warren A, Zhao F, Miao M (2015). Phylogenomics of non-model ciliates based on transcriptomic analyses. Protein Cell.

[7] Reich A, Dunn C, Akasaka K, Wessel G (2015). Phylogenomic analyses of echinodermata support the sister groups of asterozoa and echinozoa. PLoS ONE.

[8] Kutty SN, Wong WH, Meusemann K, Meier R, Cranston PS. A phylogenomic analysis of Culicomorpha (Diptera) resolves the relationships among the eight constituent families. Syst Entomol. 2018;(March):1–14.

[9] Washburn JD, Schnable JC, Conant GC, Brutnell TP, Shao Y, Zhang Y (2017). Genome-guided phylo-transcriptomic methods and the nuclear phylogentic tree of the Paniceae grasses. Sci Rep.

[10] Yang Y, Smith SA (2014). Orthology inference in nonmodel organisms using transcriptomes and low-coverage genomes: improving accuracy and matrix occupancy for phylogenomics. Mol Biol Evol.

[11] Mckain MR, Johnson MG, Urive-Convers S, Eaton D, Yang Y (2018). Practical considerations for plant phylogenomics. Appl Plant Sci.

[12] Yu X, Yang D, Guo C, Gao L (2018). Plant phylogenomics based on genome-partitioning strategies: progress and prospects. Plant Divers.

[13] Wen J, Egan AN, Dikow RB, Zimmer EA. Utility of transcriptome sequencing for phylogenetic inference and character evolution. In: Next-generation sequencing in plant systematics. 2015. p. 1–42.

[14] Whelan NV, Kocot KM, Moroz LL, Halanych KM (2015). Error, signal, and the placement of Ctenophora sister to all other animals. Proc Natl Acad Sci.

[15] Blanquart S, Lartillot N (2008). A site- and time-heterogeneous model of amino acid replacement. Mol Biol Evol.

[16] Lanfear R, Calcott B, Kainer D, Mayer C, Stamatakis A (2014). Selecting optimal partitioning schemes for phylogenomic datasets. BMC Evol Biol.

[17] Philippe H, Delsuc F, Brinkmann H, Lartillot N (2005). Phylogenomics. Annu Rev Ecol Evol Syst.

[18] Feuda R, Dohrmann M, Pett W, Philippe H, Rota-Stabelli O, Lartillot N (2017). Improved modeling of compositional heterogeneity supports sponges as sister to all other animals. Curr Biol.

[19] Wang HC, Minh BQ, Susko E, Roger AJ (2018). Modeling site heterogeneity with posterior mean site frequency profiles accelerates accurate phylogenomic estimation. Syst Biol.

[20] Zhang C, Rabiee M, Sayyari E, Mirarab S (2018). ASTRAL-III: polynomial time species tree reconstruction from partially resolved gene trees. BMC Bioinform.

[21] Liu L, Yu L, Edwards SV (2010). A maximum pseudo-likelihood approach for estimating species trees under the coalescent model. BMC Evol Biol.

[22] Borowiec ML, Lee EK, Chiu JC, Plachetzki DC (2015). Extracting phylogenetic signal and accounting for bias in whole-genome data sets supports the Ctenophora as sister to remaining Metazoa. BMC Genomics.

[23] Simion P, Phillippe H, Baurain D, Jager M, Richter DJ, Di Franco A (2017). A large and consistent phylogenomic dataset supports sponges as the sister group to all other animals. Curr Biol.

[24] Masta SE, Longhorn SJ, Boore JL (2008). Arachnid relationships based on mitochondrial genomes: asymmetric nucleotide and amino acid bias affects phylogenetic analyses. Mol Phylogenet Evol.

[25] Lasek-Nesselquist E (2012). A Mitogenomic re-evaluation of the bdelloid phylogeny and relationships among the syndermata. PLoS ONE.

[26] Wang Z, Gerstein M, Snyder M (2009). RNA-Seq: a revolutionary tool for transcriptomics. Nat Rev Genet.

[27] MacManes MD (2014). On the optimal trimming of high-throughput mRNA sequence data. Front Genet.

[28] Mbandi SK, Hesse U, Rees DJG, Christoffels A (2014). A glance at quality score: implication for de novo transcriptome reconstruction of Illumina reads. Front Genet.

[29] MacManes MD, Eisen MB (2013). Improving transcriptome assembly through error correction of high-throughput sequence reads. PeerJ.

[30] Song L, Florea L (2015). Rcorrector: efficient and accurate error correction for Illumina RNA-seq reads. Giga Sci.

[31] Le H, Schulz MH, Mccauley BM, Hinman VF, Bar-Joseph Z (2013). Probabilistic error correction for RNA sequencing. Nucleic Acids Res.

[32] MacManes MD (2018). The Oyster River Protocol: a multi-assembler and kmer approach for de novo transcriptome assembly. PeerJ.

[33] Li B, Dewey CN (2011). RSEM: accurate transcript quantification from RNA-Seq data with or without a reference genome. BMC Bioinform.

[34] Li B, Fillmore N, Bai Y, Collins M, Thomson JA, Stewart R (2014). Evaluation of de novo transcriptome assemblies from RNA-Seq data. Genome Biol.

[35] Smith-Unna R, Boursnell C, Patro R, Hibberd JM, Kelly S (2016). TransRate: reference free quality assessment of de-novo transcriptome assemblies. Genome Res.

[36] Parks MB, Wickett NJ, Alverson AJ (2017). Signal, uncertainty, and conflict in phylogenomic data for a diverse lineage of microbial eukaryotes (Diatoms, Bacillariophyta). Mol Biol Evol.

[37] Karmeinski D, Meusemann K, Goodheart JA, Schroedi M, Martynov A, Korshunova T, et al. Transcriptomics provides a robust framework for the relationships of the major clades of cladobranch sea slugs (Mollusca, Gastropoda, Heterobranchia), but fails to resolve the position of the enigmatic genus Embletonia. bioRxiv. 2020. 10.1186/s12862-021-01944-0 PMC8895541 34963462

[38] Yang Y, Smith SA (2013). Optimizing de novo assembly of short-read RNA-seq data for phylogenomics. BMC Genomics.

[39] Dunn CW, Howison M, Zapata F (2013). Agalma: an automated phylogenomics workflow. BMC Bioinform.

[40] Nguyen L, Schmidt HA, Von HA, Minh BQ (2014). IQ-TREE: a fast and effective stochastic algorithm for estimating maximum-likelihood phylogenies. Mol Biol Evol.

[41] Irisarri I, Baurain D, Brinkmann H, Delsuc F, Sire J, Kupfer A (2017). Phylotranscriptomic consolidation of the jawed vertebrate timetree. Nat Ecol Evol.

[42] Chen M-Y, Liang D, Zhang P (2017). Phylogenomic resolution of the phylogeny of laurasiatherian mammals: exploring phylogenetic signals within coding and noncoding sequences. Genome Biol Evol.

[43] Robinson DF, Foulds LR (1981). Comparison of phylogenetic trees. Math Biosci.

[44] Salichos L, Stamatakis A, Rokas A (2014). Novel information theory-based measures for quantifying incongruence among phylogenetic trees. Mol Biol Evol.

[45] Mirarab S, Reaz R, Bayzid MS, Zimmermann T, Swenson MS, Warnow T (2014). ASTRAL: genome-scale coalescent-based species tree estimation. Bioinformatics.

[46] Emms DM, Kelly S (2015). OrthoFinder: solving fundamental biases in whole genome comparisons dramatically improves orthogroup inference accuracy. Genome Biol.

[47] Emms DM, Kelly S (2019). OrthoFinder: phylogenetic orthology inference for comparative genomics. Genome Biol.

[48] Venkatesh B, Lee AP, Ravi V, Maurya AK, Lian MM, Swann JB (2014). Elephant shark genome provides unique insights into gnathostome evolution. Nature.

[49] Puig Giribets M, Pilar García Guerreiro M, Santos M, Ayala FJ, Tarrío R, Rodríguez-Trelles F (2019). Chromosomal inversions promote genomic islands of concerted evolution of Hsp70 genes in the *Drosophila**subobscura* species subgroup. Mol Ecol.

[50] Foster PG, Hickey DA (1999). Compositional bias may affect both DNA-based and protein-based phylogenetic reconstructions. J Mol Evol.

[51] Revell LJ, Harmon LJ, Collar DC (2008). Phylogenetic signal, evolutionary process, and rate. Syst Biol.

[52] Bolger AM, Lohse M, Usadel B (2014). Trimmomatic: a flexible trimmer for Illumina sequence data. Bioinformatics.

[53] Haas BJ, Papanicolaou A, Yassour M, Grabherr M, Blood PD, Bowden J (2013). De novo transcript reconstruction from RNA-Seq: reference generation and analysis with Trinity. Nat Protoc.

[54] Robertson G, Schein J, Chiu R, Corbett R, Field M, Jackman SD (2010). De novo assembly and analysis of RNA-seq data. Nat Methods.

[55] Bushmanova E, Antipov D, Lapidus A, Prjibelski AD (2019). rnaSPAdes: a de novo transcriptome assembler and its application to RNA-Seq data. Giga Sci.

[56] Li W, Godzik A (2006). Cd-hit: a fast program for clustering and comparing large sets of protein or nucleotide sequences. Bioinformatics.

[57] Simao FA, Waterhouse RM, Ioannidis P, Kriventseva EV, Zdobnov EM (2015). BUSCO: assessing genome assembly and annotation completeness with single-copy orthologs. Bioinformatics.

[58] Haas BJ, Papanicolaou A. TransDecoder. 2018. https://github.com/TransDecoder/TransDecoder/wiki.

[59] Howe KL, Contreras-moreira B, De Silva N, Maslen G, Akanni W, Allen J (2020). Ensembl Genomes 2020—enabling non-vertebrate genomic research. Nucleic Acids Res.

[60] R Core Team. R: a language and environment for statistical computing. Vienna, Austria; 2018. https://www.r-project.org/.

[61] Kocot KM, Citarella MR, Moroz LL, Halanych KM (2013). PhyloTreePruner: a phylogenetic tree-based approach for selection of orthologous sequences for phylogenomics. Evol Bioinform.

[62] Katoh K, Toh H (2010). Parallelization of the MAFFT multiple sequence alignment program. Bioinformatics.

[63] Castresana J (2000). Selection of conserved blocks from multiple alignments for their use in phylogenetic analysis. Mol Biol Evol.

[64] Talavera G, Castresana J (2007). Improvement of phylogenies after removing divergent and ambiguously aligned blocks from protein sequence alignments. Syst Biol.

[65] Dunn C, Smith S, Ryan J. Gblockswrapper. Bitbucket; 2009. https://bitbucket.org/caseywdunn/labcode/src/master/scripts_phylogenomics_21Feb2009/Gblockswrapper.

[66] Jones P, Binns D, Chang H, Fraser M, Li W, Mcanulla C (2014). InterProScan 5: genome-scale protein function classification. Bioinformatics.

[67] Alexa A, Rahnenfuhrer J. Gene set enrichment analysis with topGO. Bioconduct Improv. 2009;27.

[68] Maddison WP, Maddison DR. Mesquite: a modular system for evolutionary analysis. 2018. http://www.mesquiteproject.org.

[69] Schliep KP (2011). phangorn: phylogenetic analysis in R. Bioinformatics.

[70] Stamatakis A (2014). RAxML version 8: a tool for phylogenetic analysis and post-analysis of large phylogenies. Bioinformatics.

[71] Spillane JL, LaPolice TM, MacManes MD, Plachetzki DC (2020). Zenodo.

[72] Spillane JL. Repository for analysis of high- and low-quality transcriptome assemblies. 2019. http://github.com/jls943/quality_review. Accessed 28 July 2020.

