# Supplementary material for: Signal, bias, and the role of transcriptome assembly quality in phylogenomic inference
Source: BMC Ecol Evol. 2021 Mar 16;21:43. doi: 10.1186/s12862-021-01772-2 (PMC7968300; doi:10.1186/s12862-021-01772-2)
Supplement: Supplementary file 1 — Additional file 1: Table S1. Accession numbers and associated studies of RNA-seq read sets used in these analyses. [file 12862_2021_1772_MOESM1_ESM.docx]

| Species | Accession | Reference |
| --- | --- | --- |
| *Alligator mississippiensis* | SRR629636 | McGaugh SE, Bronikowski AM, Kuo CH, Reding DM, Addis EA, Flagel LE, Janzen FJ, Schwartz TS. Rapid molecular evolution across amniotes of the IIS/TOR network. Proceedings of the National Academy of Sciences. 2015 Jun 2;112(22):7055-60. http://dx.doi.org/10.1073/pnas.1419659112 |
| *Ambystoma mexicanum* | SRR5341572 | Nowoshilow S, Schloissnig S, Fei JF, Dahl A, Pang AW, Pippel M, Winkler S, Hastie AR, Young G, Roscito JG, Falcon F. The axolotl genome and the evolution of key tissue formation regulators. Nature. 2018 Feb;554(7690):50-5. http://dx.doi.org/10.1038/nature25458 |
| *Anas platyrhynchos* | SRR7127376 | Hérault F, Houée-Bigot M, Baéza E, Bouchez O, Esquerré D, Klopp C, Diot C. RNA-seq analysis of hepatic gene expression of common Pekin, Muscovy, mule and hinny ducks fed ad libitum or overfed. BMC genomics. 2019 Dec;20(1):1-4. http://dx.doi.org/10.1186/s12864-018-5415-1 |
| *Anolis carolinensis* | SRR391653 | Eckalbar WL, Hutchins ED, Markov GJ, Allen AN, Corneveaux JJ, Lindblad-Toh K, Di Palma F, Alföldi J, Huentelman MJ, Kusumi K. Genome reannotation of the lizard Anolis carolinensis based on 14 adult and embryonic deep transcriptomes. BMC genomics. 2013 Dec 1;14(1):49. http://dx.doi.org/10.1186/1471-2164-14-49 |
| *Astyanax mexicanus* | SRR2045431 | Pasquier J, Cabau C, Nguyen T, Jouanno E, Severac D, Braasch I, Journot L, Pontarotti P, Klopp C, Postlethwait JH, Guiguen Y. Gene evolution and gene expression after whole genome duplication in fish: the PhyloFish database. BMC genomics. 2016 Dec;17(1):1-0. http://dx.doi.org/10.1186/s12864-016-2709-z |
| *Balaenoptera acutotostrata* | SRR919296 | Yim HS, Cho YS, Guang X, Kang SG, Jeong JY, Cha SS, Oh HM, Lee JH, Yang EC, Kwon KK, Kim YJ. Minke whale genome and aquatic adaptation in cetaceans. Nature genetics. 2014 Jan;46(1):88-92. http://dx.doi.org/10.1038/ng.2835 |
| *Bufo bufo* | ERR1331718 | Jin L, Yu JP, Yang ZJ, Merilä J, Liao WB. Modulation of gene expression in liver of hibernating Asiatic Toads (Bufo gargarizans). International journal of molecular sciences. 2018 Aug;19(8):2363. http://dx.doi.org/10.3390/ijms19082363 |
| *Caecilia tentaculata* | SRR5591453 | Torres-Sánchez M, Creevey CJ, Kornobis E, Gower DJ, Wilkinson M, San Mauro D. Multi-tissue transcriptomes of caecilian amphibians highlight incomplete knowledge of vertebrate gene families. DNA Research. 2019 Feb 1;26(1):13-20. http://dx.doi.org/10.1093/dnares/dsy034 |
| *Caiman crocodilus* | ERR2198478 | No associated article. Study accession: PRJEB21261 |
| *Calidris pugnax* | ERR1018151 | Küpper C, Stocks M, Risse JE, Dos Remedios N, Farrell LL, McRae SB, Morgan TC, Karlionova N, Pinchuk P, Verkuil YI, Kitaysky AS. A supergene determines highly divergent male reproductive morphs in the ruff. Nature genetics. 2016 Jan;48(1):79-83. http://dx.doi.org/10.1038/ng.3443 |
| *Callorhinchus milii* | SRR513760 | Venkatesh B, Lee AP, Ravi V, Maurya AK, Lian MM, Swann JB, Ohta Y, Flajnik MF, Sutoh Y, Kasahara M, Hoon S. Elephant shark genome provides unique insights into gnathostome evolution. Nature. 2014 Jan;505(7482):174-9. http://dx.doi.org/10.1038/nature12826 |
| *Canis lupus*  *familiaris* | ERR1331673 | Berthelot C, Villar D, Horvath JE, Odom DT, Flicek P. Complexity and conservation of regulatory landscapes underlie evolutionary resilience of mammalian gene expression. Nature ecology & evolution. 2018 Jan;2(1):152-63. http://dx.doi.org/10.1038/s41559-017-0377-2 |
| *Dasypus novemcinctus* | SRR494766 | No associated article. Study accession: PRJNA163137 |
| *Felis catus* | ERR1331679 | Berthelot C, Villar D, Horvath JE, Odom DT, Flicek P. Complexity and conservation of regulatory landscapes underlie evolutionary resilience of mammalian gene expression. Nature ecology & evolution. 2018 Jan;2(1):152-63. http://dx.doi.org/10.1038/s41559-017-0377-2 |
| *Gadhus morhua* | SRR2045420 | Pasquier J, Cabau C, Nguyen T, Jouanno E, Severac D, Braasch I, Journot L, Pontarotti P, Klopp C, Postlethwait JH, Guiguen Y. Gene evolution and gene expression after whole genome duplication in fish: the PhyloFish database. BMC genomics. 2016 Dec;17(1):1-0. http://dx.doi.org/10.1186/s12864-016-2709-z |
| *Gallus gallus* | ERR1298598 | Kuo RI, Tseng E, Eory L, Paton IR, Archibald AL, Burt DW. Normalized long read RNA sequencing in chicken reveals transcriptome complexity similar to human. BMC genomics. 2017 Dec 1;18(1):323. http://dx.doi.org/10.1186/s12864-017-3691-9 |
| *Haplochromis burtoni* | SRR387451 | Brawand D, Wagner CE, Li YI, Malinsky M, Keller I, Fan S, Simakov O, Ng AY, Lim ZW, Bezault E, Turner-Maier J. The genomic substrate for adaptive radiation in African cichlid fish. Nature. 2014 Sep;513(7518):375-81. http://dx.doi.org/10.1038/nature13726 |
| *Homo sapiens* | SRR5576267 | Kim DS, Ryu JW, Son MY, Oh JH, Chung KS, Lee S, Lee JJ, Ahn JH, Min JS, Ahn J, Kang HM. A liver‐specific gene expression panel predicts the differentiation status of in vitro hepatocyte models. Hepatology. 2017 Nov;66(5):1662-74. http://dx.doi.org/10.1002/hep.29324 |
| *Ictalurus punctatus* | SRR917955 | Liu S, Wang X, Sun F, Zhang J, Feng J, Liu H, Rajendran KV, Sun L, Zhang Y, Jiang Y, Peatman E. RNA-Seq reveals expression signatures of genes involved in oxygen transport, protein synthesis, folding, and degradation in response to heat stress in catfish. Physiological genomics. 2013 Jun 15;45(12):462-76. http://dx.doi.org/10.1152/physiolgenomics.00026.2013 |
| *Latimeria menadoensis* | SRR576100 | Pallavicini A, Canapa A, Barucca M, Alfőldi J, Biscotti MA, Buonocore F, De Moro G, Di Palma F, Fausto AM, Forconi M, Gerdol M. Analysis of the transcriptome of the Indonesian coelacanth Latimeria menadoensis. BMC genomics. 2013 Dec 1;14(1):538. http://dx.doi.org/10.1186/1471-2164-14-538 |
| *Lepidophyma flavimaculatum* | DRR034613 | No associated article. Study accession: PRJDB3883 |
| *Lepisosteus oculatus* | SRR1287992 | No associated article. Study accession: PRJNA247500 |
| *Lethenteron camtschaticum* | SRR3223459 | Du K, Zhong Z, Fang C, Dai W, Shen Y, Gan X, He S. Ancient duplications and functional divergence in the interferon regulatory factors of vertebrates provide insights into the evolution of vertebrate immune systems. Developmental & Comparative Immunology. 2018 Apr 1;81:324-33. http://dx.doi.org/10.1016/j.dci.2017.12.016 |
| *Lissotriton montandoni* | SRR3299753 | Stuglik MT, Babik W. Genomic heterogeneity of historical gene flow between two species of newts inferred from transcriptome data. Ecology and evolution. 2016 Jul;6(13):4513-25. http://dx.doi.org/10.1002/ece3.2152 |
| *Notamacropus eugenii* | DRR013408, DRR013409, DRR013410 | Deakin JE. Genome Sequence of an Australian Kangaroo, Macropus eugenii. eLS. 2013. http://dx.doi.org/10.1186/gb-2011-12-8-r81 |
| *Notechis scutatus* | SRR519122 | No associated article. Study accession: PRJNA170152 |
| *Oophaga sylvatica* | SRR9120851 | Caty SN, Alvarez-Buylla A, Byrd GD, Vidoudez C, Roland AB, Tapia EE, Budnik B, Trauger SA, Coloma LA, O'Connell LA. Molecular physiology of chemical defenses in a poison frog. Journal of Experimental Biology. 2019 Jun 15;222(12):jeb204149. http://dx.doi.org/10.1242/jeb.204149 |
| *Oryctolagus cuniculus* | ERR1331669 | Berthelot C, Villar D, Horvath JE, Odom DT, Flicek P. Complexity and conservation of regulatory landscapes underlie evolutionary resilience of mammalian gene expression. Nature ecology & evolution. 2018 Jan;2(1):152-63. http://dx.doi.org/10.1038/s41559-017-0377-2 |
| *Parus major* | SRR1847228 | Charmantier A, Gienapp P. Climate change and timing of avian breeding and migration: evolutionary versus plastic changes. Evolutionary Applications. 2014 Jan;7(1):15-28. http://dx.doi.org/10.1111/eva.12126 |
| *Pelodiscus sinensis* | SRR6157006 | Zeng D, Li X, Wang XQ, Xiong G. Development of SNP markers associated with growth-related genes of Pelodiscus sinensis. Conservation Genetics Resources. 2020 Mar;12(1):87-92. http://dx.doi.org/10.1007/s12686-018-1065-5 |
| *Pelusios castaneus* | SRR629649 | McGaugh SE, Bronikowski AM, Kuo CH, Reding DM, Addis EA, Flagel LE, Janzen FJ, Schwartz TS. Rapid molecular evolution across amniotes of the IIS/TOR network. Proceedings of the National Academy of Sciences. 2015 Jun 2;112(22):7055-60. http://dx.doi.org/10.1073/pnas.1419659112 |
| *Protopterus sp.* | ERR2202465 | Chana-Muñoz A, Jendroszek A, Sønnichsen M, Wang T, Ploug M, Jensen JK, Andreasen PA, Bendixen C, Panitz F. Origin and diversification of the plasminogen activation system among chordates. BMC evolutionary biology. 2019 Dec 1;19(1):27. http://dx.doi.org/10.1186/s12862-019-1353-z |
| *Rana pipiens* | SRR1185245 | Christenson MK, Trease AJ, Potluri LP, Jezewski AJ, Davis VM, Knight LA, Kolok AS, Davis PH. De novo assembly and analysis of the northern leopard frog Rana pipiens transcriptome. Journal of genomics. 2014;2:141. http://dx.doi.org/10.7150/jgen.9760 |
| *Rhinella marina* | SRR6311453 | Russo AG, Eden JS, Tuipulotu DE, Shi M, Selechnik D, Shine R, Rollins LA, Holmes EC, White PA. Viral discovery in the invasive Australian cane toad (Rhinella marina) using metatranscriptomic and genomic approaches. Journal of virology. 2018 Sep 1;92(17). http://dx.doi.org/10.1128/JVI.00768-18 |
| *Rhinolophus sinicus* | SRR2273875 | Dong D, Lei M, Hua P, Pan YH, Mu S, Zheng G, Pang E, Lin K, Zhang S. The genomes of two bat species with long constant frequency echolocation calls. Molecular Biology and Evolution. 2016 Oct 26:msw231. http://dx.doi.org/10.1093/molbev/msw231 |
| *Squalus acanthias* | ERR1525379 | Chana-Munoz A, Jendroszek A, Sønnichsen M, Kristiansen R, Jensen JK, Andreasen PA, Bendixen C, Panitz F. Multi-tissue RNA-seq and transcriptome characterisation of the spiny dogfish shark (Squalus acanthias) provides a molecular tool for biological research and reveals new genes involved in osmoregulation. PloS one. 2017 Aug 23;12(8):e0182756. http://dx.doi.org/10.1371/journal.pone.0182756 |
| *Takifugu rubripes* | SRR1005688 | No associated article. Study accession: PRJNA222262 |
| *Trachemys scripta* | ERR2198830 | Chana-Muñoz A, Jendroszek A, Sønnichsen M, Wang T, Ploug M, Jensen JK, Andreasen PA, Bendixen C, Panitz F. Origin and diversification of the plasminogen activation system among chordates. BMC evolutionary biology. 2019 Dec 1;19(1):27. http://dx.doi.org/10.1186/s12862-019-1353-z |
