# Supplementary figures and images for: Signal, bias, and the role of transcriptome assembly quality in phylogenomic inference
Source: BMC Ecol Evol. 2021 Mar 16;21:43. doi: 10.1186/s12862-021-01772-2 (PMC7968300; doi:10.1186/s12862-021-01772-2)

**A**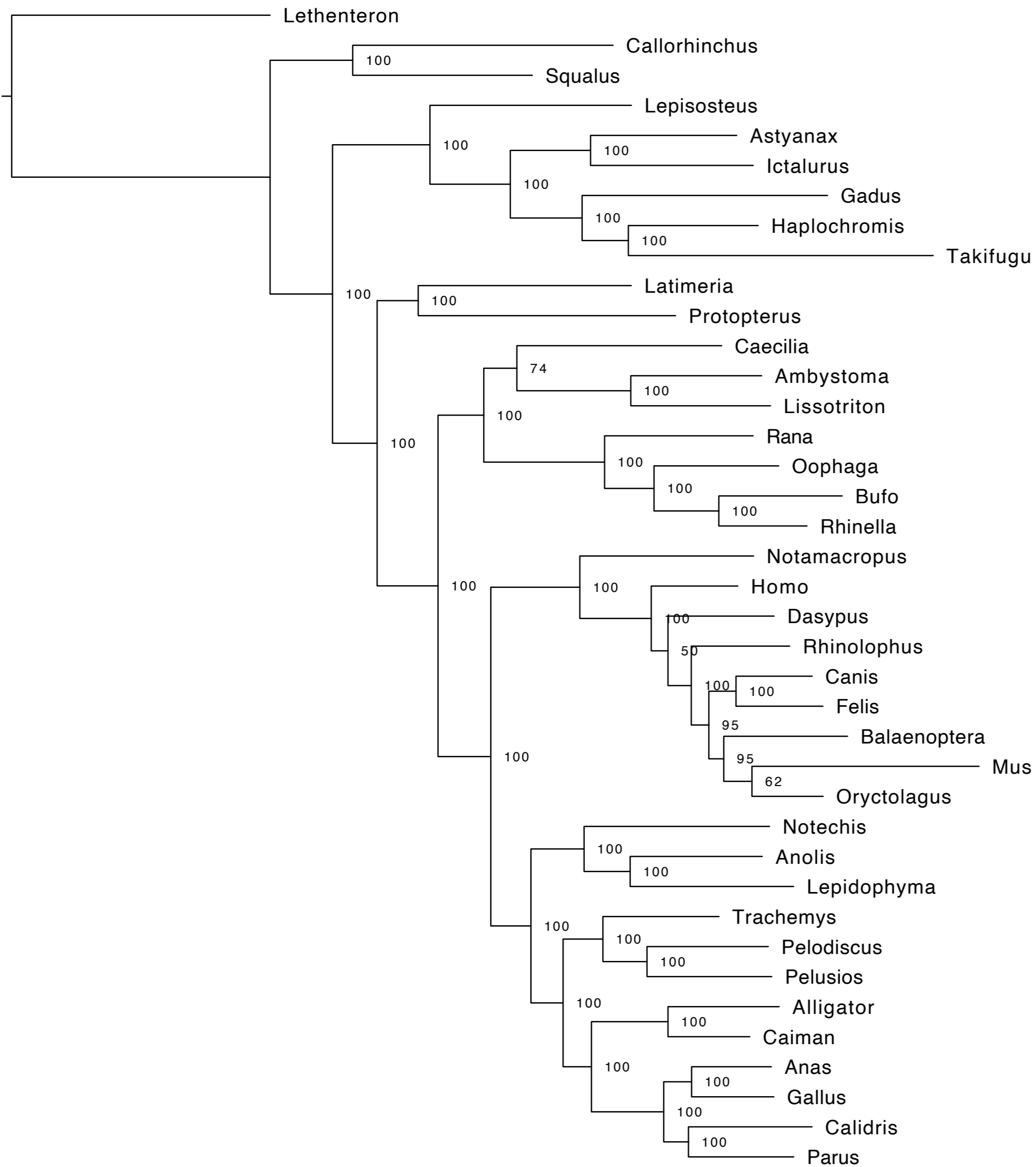**B**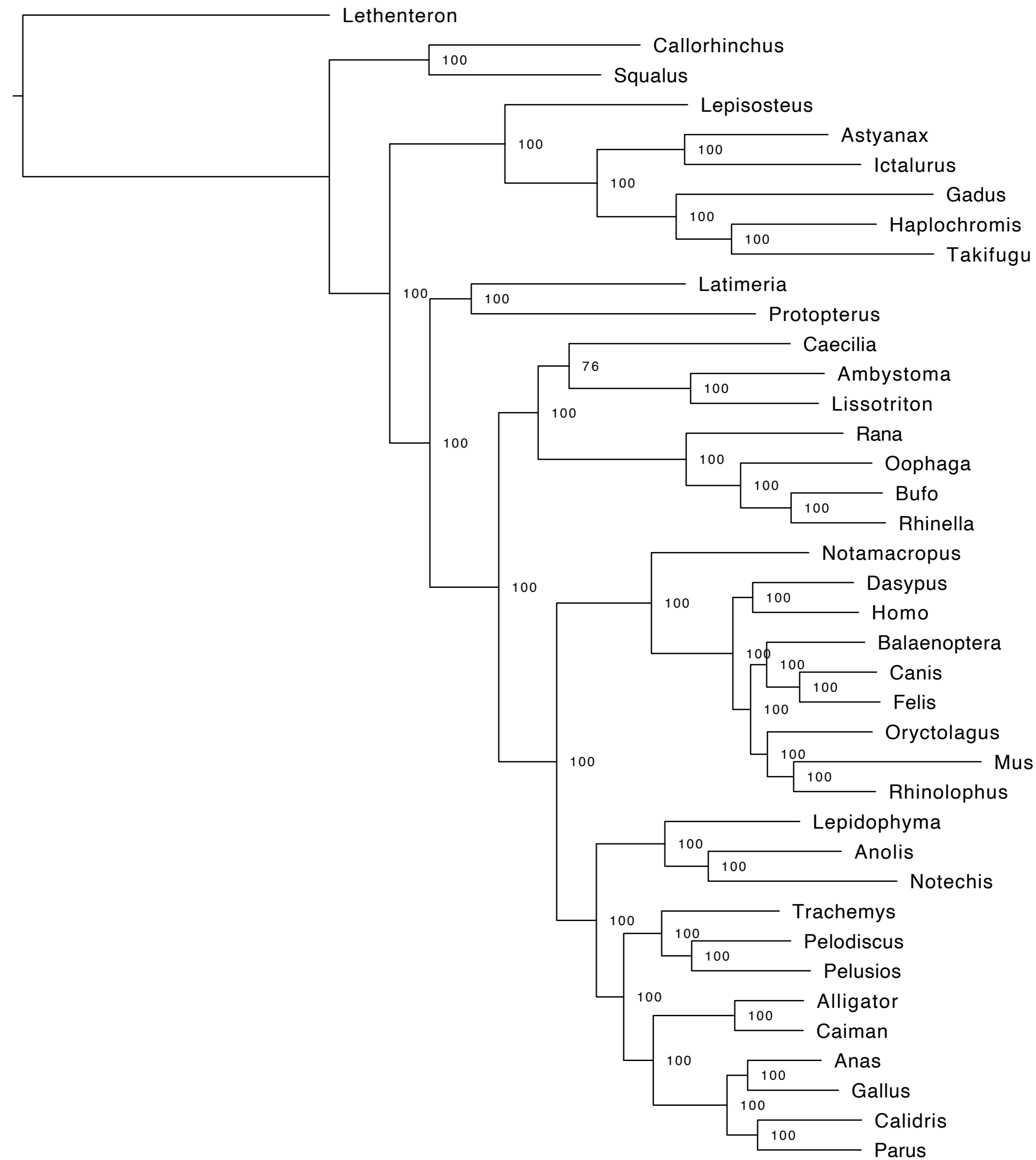

Supplement: Supplementary file 2 — Additional file 2: Figure S1. Phylogenetic trees created using the 332 data partitions shared between the two datasets and concatenation methods do not resolve the accepted craniate phylogeny but produce differing topologies. The trees were built in IQ-TREE using an LG model and nodes are labeled with ultrafast bootstrap approximated branch supports using the “-bnni” (a hill-climbing nearest neighbor interchange search) to reduce the impact of severe model violations. A: Phylogenetic tree for the low-quality dataset. B: Phylogenetic tree for the high-quality dataset. [file 12862_2021_1772_MOESM2_ESM.pdf]
